# Improved Outcome with Early Rifampicin Combination Treatment in Methicillin-Sensitive *Staphylococcus aureus* Bacteraemia with a Deep Infection Focus – A Retrospective Cohort Study

**DOI:** 10.1371/journal.pone.0122824

**Published:** 2015-04-13

**Authors:** Erik Forsblom, Eeva Ruotsalainen, Asko Järvinen

**Affiliations:** Division of Infectious Diseases, Department of Medicine, Helsinki University Central Hospital, Helsinki, Finland; Cleveland Clinic, UNITED STATES

## Abstract

**Introduction:**

Rifampicin has been used as adjunctive therapy in *Staphylococcus aureus* bacteraemia (SAB) with a deep infection focus. However, data for prognostic impact of rifampicin therapy is unestablished including the optimal initiation time point. We studied the impact of rifampicin therapy and the optimal initiation time for rifampicin treatment on prognosis in methicillin-sensitive *S*. *aureus* bacteraemia with a deep infection.

**Methods:**

Retrospective, multicentre study in Finland including 357 SAB patients with a deep infection focus. Patients with alcoholism, liver disease or patients who died within 3 days were excluded. Patients were categorised according to duration of rifampicin therapy and according to whether rifampicin was initiated early (within 7 days) or late (7 days after) after the positive blood cultures. Primary end point was 90 days mortality.

**Results:**

Twenty-seven percent of patients received no rifampicin therapy, 14% received rifampicin for 1-13 days whereas 59% received rifampicin ≥14 days. The 90 day mortality was; 26% for patients treated without rifampicin, 16% for rifampicin therapy of any length and 10% for early onset rifampicin therapy ≥14 days. Lack of rifampicin therapy increased (OR 1.89, p=0.026), rifampicin of any duration decreased (OR 0.53, p=0.026) and rifampicin therapy ≥14 days with early onset lowered the risk for a fatal outcome (OR 0.33, p<0.01) during 90 days follow-up.

**Conclusion:**

Rifampicin adjunctive therapy for at least 14 days and initiated within 7 days of positive blood culture associated with improved outcome among SAB patients with a deep infection.

## Introduction

*Staphylococcus aureus* is one of the most common pathogens in bacteraemic infections [1–3]. However, treatment results in *S*. *aureus* bacteraemia (SAB) have not been considerably improved despite the introduction of new antibiotics [1]. Complications and mortality in SAB have been related to methicillin-resistance [2,3] and haematogenous dissemination resulting in deep infection foci like endocarditis [3,4]. In recent studies, deep infection foci have been found in up to 80% of SAB patients [5,6]. Adjunctive rifampicin therapy has been explored as a way of improving treatment results in SAB with deep infections [5,7–15] whereas only few studies have evaluated the impact of adjunctive rifampicin therapy in SAB without a deep infection focus [8,9]. Rifampicin may be potentially valuable in *S*. *aureus* infections due to its bactericidal nature, high anti-staphylococcal activity and good biofilm penetrating capability [16–18]. Conventionally *S*. *aureus* has been regarded primarily as an extracellular bacterium although much data point towards possibilities of intracellular survival [19,20]. Intracellular survival has been suggested to enhance haematogenous dissemination and to result in subpopulation formation with reduced antibiotic susceptibility and relapse of *S*. *aureus* infection [19–23]. Recently it was proposed that the capability of rifampicin to achieve high intracellular concentrations may be essential for eradication of intra-leucocyte *S*. *aureus* infections [21].

In most studies, rifampicin has been combined to a staphylococcal penicillin [7–9,24], a glycopeptide (vancomycin) [8,9,25,26] or to a fluoroquinolone [10–15] in patients with chronic osteomyelitis, foreign body infections, endocarditis or other deep seated abscesses. These studies have been small in size including only from 14 to maximally 105 patients [7–15,24] and few studies only have been controlled and randomized [8–11,25]. Accordingly, statistically significantly higher cure rate with rifampicin treatment has been observed only in a few studies [5,8,11,14]. Recently, a stratified meta-analysis regarding *Staphylococcus aureus* bacteraemic patients only demonstrated a non-significant reduction in infection related deaths as a result of adjunctive rifampicin therapy [27].

Monotherapy with rifampicin results in rapid resistance development and combination therapy is a prerequisite for use of rifampicin in SAB [28,29]. Resistance development has been observed in studies with methicillin-resistant *S*. *aureus* (MRSA) or heteroresistant vancomycin-intermediate *S*. *aureus* (hVISA) [25,26,30,31]. In these studies, no positive prognostic effect of rifampicin was observed. In one study, 56% of patients developed rifampicin resistance and in each case the patient was bacteraemic at rifampicin initiation whereas none of the patients with negative blood cultures at rifampicin initiation developed resistance [26]. In this study, adjunctive rifampicin was associated with prolonged bacteraemia and poorer outcome which resulted in recommendations to commence rifampicin first after clearance of bacteraemia [26]. A large prospective study, primarily investigating the beneficial effect of a levofloxacin combination therapy in methicillin-sensitive *S*. *aureus* (MSSA) bacteraemia, produced a *post hoc* analysis with 331 patients on the effect of rifampicin on mortality. Rifampicin adjunctive therapy significantly improved outcome in patients with a deep infection focus [5]. This study was, however, not randomized, did not give an answer as to when rifampicin treatment should be started and did not identify the minimum duration of rifampicin therapy needed for a positive prognostic impact. Therefore, we found it interesting to study if rifampicin including combination therapy would affect the outcome in SAB patients with a deep infection focus and how the time point of rifampicin onset would affect the results in a larger patient material.

## Materials and Methods

### Patients and data collection

Altogether 617 adult patients with at least one positive blood culture for *S*. *aureus* were identified. When accounting for the exclusion and inclusion criteria a total of 357 patients with a deep infection focus were accepted for this retrospective study. Part of the patients had participated in our earlier prospective multicentre study including all five university and seven central hospitals in Finland during January 1999 to May 1999 and January 2000 to August 2002 [5]. The material was further extended with all SAB cases identified retrospectively who were not included into the previous prospective study between 1999–2002 [5] and all SAB patients between 2006–2007 from Helsinki University Central Hospital [6]. Two time-periods were included to exclude any unknown temporary differences in personnel or treatment practices. Data collection included age, gender, underlying diseases, McCabe’s classification on underlying diseases [32], medication, severe sepsis, septic shock and intensive care unit (ICU) treatment. Any infectious diseases specialist (IDS) consultation was documented. Infection foci documentation were based on clinical suspicion solely or verified by bacteriological, radiological or pathological findings. All antibiotic therapy including length and route of administration were documented. Patients were followed for 90 days and primary outcome was mortality at 90 days.

### Exclusion criteria

No MRSA cases were included. Patients suffering from alcoholism and acute or chronic liver diseases were excluded. These conditions may induce elevated liver enzymes and liver failure during rifampicin therapy. Thus these conditions carry a risk for a statistical bias as patients with these conditions are unlikely to receive adjunctive rifampicin therapy. The mean time-lapse between blood culture collection and positive blood culture results was 3 days and thus patients with a fatal outcome within 3 days of blood culture collection were excluded in order to allow for a fatal outcome before positive blood culture results and the possibility to initiate rifampicin therapy.

### Ethics statement

The trial was approved by *The institutional review board of Helsinki University Central Hospital* and *The Ethical committee of Helsinki University Central Hospital*. A written informed consent was provided by each patient.

### Definitions

Modified Duke criteria were used to define endocarditis [33]. Deep infection foci included pneumonia, endocarditis, purulent arthritis, osteomyelitis, deep-seated abscess and any foreign-body infection. IDS consultation within 7 days of positive blood cultures were categorized as informal telephone consultation or formal bedside consultation [6]. Severe sepsis and septic shock was classified as sepsis in combination with hypotension, hypoperfusion, or organ failure [34].

### The length of antibiotic therapy

Patients were categorized according to duration of rifampicin therapy into four groups i) no rifampicin therapy, ii) short rifampicin therapy (1–13 days), iii) long rifampicin therapy (≥14 days) and iv) rifampicin therapy of any length (including all patients having received rifampicin therapy). Patients with rifampicin therapy for at least 14 days were further classified according to whether onset of rifampicin therapy took place within 7 days (early onset) or 7 days after (late onset) the positive blood culture for *S*. *aureus*. Rifampicin was administered 450 mg once daily for patients under 50 kg and 600 mg once daily for patients over 50 kg in weight. The standard antibiotic therapy was either cloxacillin, cefuroxime, ceftriaxone, vancomycin or clindamycin. Fluoroquinolone and aminoglycoside served as additional antibiotic therapy. Fluoroquinolone therapy included either levo-, moxi- or ciprofloxacin. Aminoglycoside therapy included either tobramycin or gentamicin.

### Statistical analysis

Categorical variables were compared with Pearson’s *X* ^*2*^-test and odd ratios [OR] of 95% confidence intervals [CI] were calculated. Univariate factors with p<0.1 were entered into Cox regression model to estimate factors predicting 90-day mortality. All tests were two-tailed with p<0.05 as significant. SPSS version 12.0 [SPSS Inc., Chicago, IL, USA] was used.

## Results

### Patient characteristics

**Fig 1:**
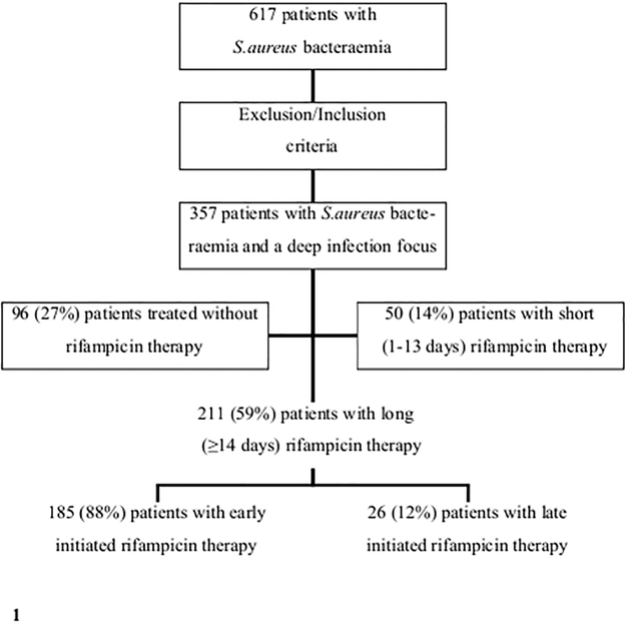
Study profile. Originally 617 patients with methicillin-sensitive *S*. *aureus* bacteraemia were identified. Patients with methicillin-resistant *S*. *aureus* bacteraemia were not included in the study (n = 6). The exclusion criteria were: patients with fatal outcome within 3 days of positive blood cultures, patients with alcoholism, acute or chronic liver diseases and patients without a deep infection foci. In total, 260 patients were excluded and altogether 357 patients were left for the analyses. Early initiation of rifampicin therapy was defined as an initiation within 7 days of positive blood cultures whereas late initiation of rifampicin therapy was defined as an initiation 7 days past positive blood cultures.

Altogether 617 SAB patients were identified. Patients with alcoholism or acute or chronic liver disease and fatal outcome within three days were excluded and only patients with a deep infection focus were included. Furthermore, patients with MRSA bacteraemia were not included in the study (n = 6). In total, 260 patients were excluded and altogether 357 patients were left for the analyses (Fig 1). Ninety-six (27%) of all 357 patients were treated without adjunctive rifampicin, 50 (14%) patients received adjunctive rifampicin for 1–13 days (short therapy) whereas 211 (59%) received adjunctive rifampicin for ≥14 days (long therapy) (Table 1). Table 1 present basic characteristics between the various treatment groups. Patients treated with short rifampicin therapy were more often males (OR 2.11, p<0.05) and had less often healthcare-associated SAB (OR 0.41, p<0.05) as compared to patients treated without rifampicin. No significant difference regarding age, chronic diseases according to McCabe′s classification and severity of illness at blood culture collection were observed between patients treated with short rifampicin therapy or no rifampicin therapy. The only difference between patients with short and long rifampicin treatment was the lower number of patients with septic shock at blood culture collection time point in the long rifampicin treatment group (1% vs. 6%, p<0.05). However, the total amount of patients with septic shock at blood culture collection time point was only 6 (1.7%) (Table 1).

**Table 1:** Characteristics of 357 patients with methicillin-sensitive *S*. *aureus* bacteraemia (SAB) and a deep infection focus categorized according to duration of rifampicin therapy.

	Rifampicin therapy			Short therapy vs. no therapy		Long therapy vs. short therapy	
Variables	No therapy n = 96 (27)	Short therapy n = 50 (14)	Long therapy n = 211 (59)	OR (95% CI)	p- value	OR (95% CI)	p- value
**Male sex**	46 (48)	33 (66)	139 (66)	2.11(1.04–4.29)	<0.05	0.99(0.52–1.91)	NS
**Age >60 years**	60 (63)	26 (52)	105 (50)	0.65(0.33–1.29)	NS	0.91(0.49–1.69)	NS
**Healthy-nonfatal disease** ^**A**^	62 (65)	34 (68)	165 (78)	1.17(0.56–2.41)	NS	1.69(0.86–3.33)	NS
**Ultimately-rapidly fatal disease** ^**A**^	34 (35)	16 (32)	46 (22)	0.86(0.42–1.78)	NS	0.59(0.30–1.17)	NS
**Healthcare-associated SAB**	68 (71)	25 (50)	107 (51)	0.41(0.20–0.84)	<0.05	1.03(0.56–1.91)	NS
**Intensive care unit** ^**B**^	14 (15)	8 (16)	38 (18)	1.12(0.43–2.87)	NS	1.15(0.50–2.65)	NS
**Severe sepsis** ^**B**^	4 (4)	5 (10)	14 (7)	2.56(0.65–9.98)	NS	0.64(0.22–1.87)	NS
**Septic shock** ^**B**^	1 (1)	3 (6)	2 (1)	5.71(0.58–56.6)	NS	0.19(0.03–1.14)	<0.05
**Formal bedside IDS consultation** ^**C**^	84 (88)	49 (98)	194 (92)	7.00(0.88–55.5)	<0.05	0.23(0.03–1.79)	NS
**Informal telephone IDS consultation** ^**C**^	9 (9)	0	14 (7)	—	—	—	—
**Cloxacillin,** standard antibiotic	57 (59)	35 (70)	116 (55)	1.59(0.77–3.31)	NS	0.52(0.27–1.02)	NS
**Cefuroxime**, standard antibiotic	24 (25)	13 (26)	61 (29)	1.05(0.48–2.31)	NS	1.16(0.58–2.33)	NS
**Ceftriaxone**, standard antibiotic	8 (8)	0	16 (8)	—	—	—	—
**Vancomycin**, standard antibiotic	1 (1)	1 (2)	5 (2)	1.94(0.12–31.7)	NS	1.19(0.14–10.4)	NS
**Clindamycin**, standard antibiotic	3 (3)	0	3 (1)	—	—	—	—
**Fluoroquinolone**, additional antibiotic^D^	59 (61)	26 (52)	101 (48)	0.68(0.34–1.36)	NS	0.85(0.46–1.57)	NS
**Aminoglycoside**, additional antibiotic ^E^	9 (9)	10 (20)	42 (20)	2.42(0.91–6.41)	NS	0.99(0.46–2.15)	NS
**Endocarditis** ^**F**^	8 (8)	12 (24)	42 (20)	3.47(1.31–9.18)	<0.01	0.79(0.38–1.64)	NS
**Deep-seated abscess** ^**F**^	39(41)	23 (46)	117 (55)	1.25(0.63–2.48)	NS	1.46(0.79–2.71)	NS
**Foreign body infection** ^**F**^	29 (30)	8 (16)	48 (23)	0.44(0.18–1.05)	NS	1.55(0.68–3.52)	NS
**Septic arthritis or osteomyelitis** ^**F**^	39 (41)	28 (56)	98 (46)	1.86(0.93–3.71)	NS	0.69(0.37–1.28)	NS
**SAB relapse** ^**G**^	2 (2)	0	2 (1)	—	—	—	—

Patients with alcoholism, acute or chronic liver diseases, lack of deep infection foci, MRSA bacteraemia (n = 6) or a fatal outcome within 3 days have been excluded. Values are expressed as n (%). NS = non-significant. Short rifampicin therapy 1–13 days. Long rifampicin therapy ≥14 days.

^A^ Classification according to McCabe and Jackson [32].

^B^ Severity of illness at blood culture collection time point.

^C^ Infectious diseases specialist (IDS) consultation.

^D^ Fluoroquinolone: levo-, moxi- or ciprofloxacin.

^E^ Aminoglycoside: tobramycin or gentamicin.

^F^ Deep infection focus within 90 days follow-up.

^G^ SAB relapse within 90 days of follow-up.

### Antibiotic therapy

Altogether 99% of patients had intravenous antibiotic therapy that was effective *in vitro* against the *S*. *aureus* blood isolate. Vancomycin was given empirically in only 2% of cases. No difference was observed in the standard antibiotic therapy given after the blood culture results between the various rifampicin treatment groups (Table 1). Antistaphylococcal penicillin (cloxacillin) was given to 208 (58%) patients whereas a non cell-wall active agent (clindamycin) was used in only 1.7% of patients. Fluoroquinolone (levo-, moxi- or ciprofloxacin) as an additional antibiotic was provided to 186 (52%) of patients and no significant difference between the patients with no rifampicin therapy, short or long rifampicin therapy was observed. No significant difference in aminoglycoside treatment was observed between the various treatment groups (Table 1).

### Deep infection foci and consultations

The vast majority of deep infection foci were verified by bacteriological, radiological or pathological findings whereas only a few pneumonia cases (n = 5) were based on a clinical suspicion only. Patients treated with short rifampicin therapy received more often bedside IDS consultation (OR 7.00, p <0.05) and had more endocarditis diagnosed (OR 3.47, p<0.01) as compared to patients treated without rifampicin. When comparing patients with short rifampicin therapy to patients with long rifampicin therapy no significant difference in distribution of bedside IDS consultation or deep infection foci were observed. Altogether only 6% of the patients received informal telephone IDS (Table 1).

### Outcome

**Fig 2:**
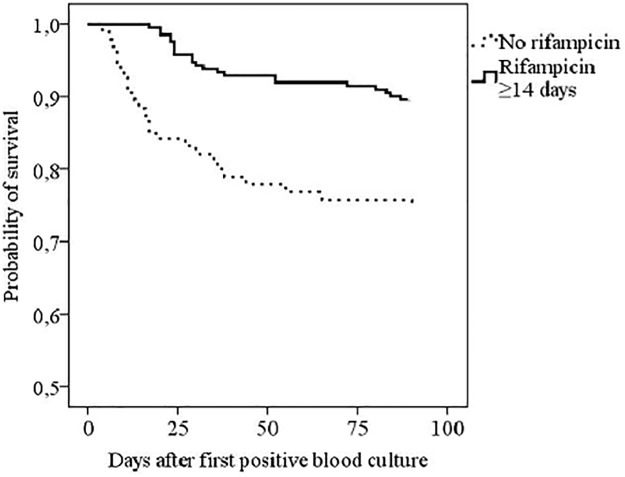
Kaplan-Meier analysis of 90 days survival in 307 *Staphylococcus aureus* bacteraemia patients with a deep infection focus according to rifampicin therapy (Log-Rank 0.000). The exclusion criteria were: patients with fatal outcome within 3 days of positive blood cultures, patients with alcoholism, acute or chronic liver diseases and patients without a deep infection foci. Patients with methicillin-resistant *S*. *aureus* bacteraemia were not included in the study (n = 6).

The total case fatality in 357 patients at 90 days was 18%. When comparing rifampicin therapy of any length to no rifampicin therapy, no significant difference in case fatality was seen at 28 days (11% vs. 17%, p = 0.130) whereas patients with rifampicin treatment had significantly lower mortality at 60 days (13% vs. 23%, p = 0.027) and at 90 days (16% vs. 26%, p = 0.026) (Table 2, Fig 2).

**Table 2:** Bacteraemic relapse and outcome of 357 patients with methicillin-sensitive *S*. *aureus* bacteraemia (SAB) and a deep infection focus categorized according to adjunctive rifampicin therapy.

	Rifampicin therapy		Any length of rifampicin therapy vs. no rifampicin therapy	
Variables	No therapy n = 96 (27%)	Therapy of any length n = 261 (73%)	OR (95% CI)	p- value
**SAB relapse** ^**A**^	2 (2)	2 (<1)	0.40 (0.06–2.89)	0.349
**Mortality**, at 28 days	16 (17)	28 (11)	0.60 (0.31–1.17)	0.130
**Mortality**, at 60 days	22 (23)	35 (13)	0.51 (0.28–0.93)	0.027
**Mortality**, at 90 days	25 (26)	41 (16)	0.53 (0.30–0.93)	0.026

Patients with alcoholism, acute or chronic liver diseases, lack of deep infection foci, MRSA bacteraemia (n = 6) or a fatal outcome within 3 days have been excluded. Values are expressed as n (%).

^A^ SAB relapse within 90 days follow-up

The prognostic impact of rifampicin therapy and early or late onset of rifampicin therapy was evaluated by univariate analysis and Cox regression analysis (Table 3). In univariate analysis, parameters with positive prognostic impact were McCabes′s healthy-nonfatal underlying conditions (OR 0.18, p<0.001), rifampicin therapy of any duration (OR 0.53, p = 0.026), rifampicin therapy ≥14 days with early onset (OR 0.28, p<0.001) and formal bedside IDS consultation (OR 0.41, p = 0.029). Age >60 years (OR 3.02, p<0.001), McCabe′s ultimately-rapidly fatal underlying diseases (OR 5.45, p<0.001), healthcare-associated SAB (OR 2.23, p = 0.006), ICU treatment (OR 2.22, p = 0.013), severe sepsis (OR 3.12, p = 0.008), endocarditis (OR 2.11, p = 0.019), lack of rifampicin therapy (OR 1.89, p = 0.026) and informal IDS telephone consultation (OR 3.12, p = 0.008) were associated with negative prognosis. Gender, SAB relapse, fluoroquinolone therapy, aminoglycoside therapy and late onset of rifampicin therapy had no significant prognostic impact in univariate analysis (Table 3). In Cox regression analysis, the parameters age >60 years (OR 2.00, p = 0.017), severe sepsis (OR 3.64, p = 0.01), endocarditis (OR 2.68, p = 0.01) and informal telephone consultation (OR 2.11, p = 0.04) had a negative prognostic impact whereas McCabes′s healthy-nonfatal underlying condition (OR 0.23, p<0.01) and rifampicin therapy ≥14 days with early onset (OR 0.33, p<0.01) were markers of positive prognosis (Table 3). In order to investigate whether patients who died early were sicker, and thus more likely to be treated without rifampicin, the analyses of Table 3 were repeated by excluding patients with a fatal outcome within 21 days. The results of this Cox regression analysis resembled those of Table 3 with a positive prognostic impact of early onset of rifampicin therapy for ≥14 days (OR 0.48, p<0.01).

**Table 3:** Cox regression analysis for prognostic factors according to 90-day mortality in 357 patients with methicillin-sensitive *S*. *aureus* bacteraemia (SAB) with at least one deep infection focus.

	Univariate analysis				Cox regression analysis ^1^	
	Survived N = 291 (82)	Died N = 66 (18)	OR (95% CI)	p- value	OR (95% CI)	p- value
**Male sex**	181 (62)	37 (56)	0.78(0.45–1.33)	NS	—	—
**Age >60 years**	142 (49)	49 (74)	3.02(1.66–5.49)	<0.001	2.00(1.13–3.54)	0.017
**Healthy-nonfatal disease** ^**A**^	233 (80)	28 (42)	0.18(0.10–0.32)	<0.001	0.23(0.14–0.39)	<0.01
**Ultimately-rapidly fatal disease** ^**A**^	58 (20)	38 (58)	5.45(3.09–9.61)	<0.001	—	—
**Healthcare associated SAB**	153 (53)	47 (71)	2.23(1.25–3.99)	0.006	—	—
**Intensive care unit** ^**B**^	42 (14)	18 (27)	2.22(1.18–4.19)	0.013	—	—
**Severe sepsis** ^**B**^	14 (5)	9 (14)	3.12(1.29–7.57)	0.008	3.64(1.76–7.53)	0.01
**Endocarditis** ^**C**^	44 (15)	18 (27)	2.11(1.12–3.95)	0.019	2.68(1.53–4.71)	0.01
**Fluoroquinolone therapy** ^**D**^	156 (54)	30 (45)	0.72(0.42–1.23)	NS	—	—
**Aminoglucoside therapy**	45 (15)	16 (24)	1.75(0.92–3.34)	NS	—	—
**Lack of rifampicin therapy**	71 (24)	25 (38)	1.89(1.07–3.32)	0.026	—	—
**Rifampicin therapy of any duration** ^**E**^	220 (76)	41 (62)	0.53(0.30–0.93)	0.026	—	—
**Rifampicin therapy ≥14 days, early onset** ^**F**^	167 (57)	18 (27)	0.28(0.15–0.50)	<0.001	0.33(0.19–0.57)	<0.01
**Rifampicin therapy ≥14 days, late onset** ^**G**^	22 (8)	4 (6)	0.79(0.26–2.37)	NS	—	—
**Bedside IDS consultation** ^**H**^	271 (93)	56 (84)	0.41(0.18–0.93)	0.029	—	—
**Telephone IDS consultation** ^**H**^	14 (5)	9 (14)	3.12(1.29–7.57)	0.008	2.11(1.01–4.42)	0.04

Patients with alcoholism, acute or chronic liver diseases, lack of deep infection foci, MRSA bacteraemia (n = 6) or a fatal outcome within 3 days have been excluded. Values are expressed as N (%) and odds ratio (OR) for fatal outcome within 90 days. NS = non-significant.

^1^ Cox regression analysis results are shown only for parameters with significant prognostic impact (p<0.05).

^A^ Classification according to McCabe and Jackson [32].

^B^ Severity of illness at blood culture collection time point

^C^ Endocarditis diagnosed within 90 days follow-up.

^D^ Fluoroquinolone: levo-, moxi- or ciprofloxacin.

^E^ Including all patients with rifampicin therapy of any length.

^F^ Early onset i.e. onset within 7 days of positive blood culture.

^G^ Late onset i.e. onset 7 days after positive blood culture.

^H^ Infectious diseases specialist (IDS) consultation

## Discussion

The main finding of this study was a potential positive prognostic impact due to adjunctive rifampicin therapy in patients with a deep infection focus in methicillin-sensitive SAB. During the follow-up time period of 90 days, the mortality among patients treated with adjunctive rifampicin therapy for at least 14 days started during the first week of the positive blood culture was ten percent whereas the mortality for patients treated without rifampicin therapy during the corresponding time period was twenty-six percent. Early onset adjunctive rifampicin therapy for at least 14 days decreased the risk of fatal outcome to one third in Cox regression analysis.

Our study demonstrating significantly improved treatment results with rifampicin combination therapy in SAB with a deep infection focus is in line with previous studies [8,11,14] and studies showing a non-significant tendency towards better treatment result with rifampicin [7,9,10,15]. In common to most of these studies has been a low MRSA prevalence. In contrast to many previous studies, we only analysed mortality as the main outcome parameter. Rifampicin adjunctive therapy did not influence SAB relapse although this conclusion has to be made with a great caution due to the low occurrence of SAB relapses in our material. Early mortality, i.e. mortality during the 3 first days, was excluded from the analysis to be able to see the unbiased effect of rifampicin therapy on outcome.

Studies of rifampicin combination therapy in SAB with high MRSA prevalence (76–100%) have reported poorer clinical outcome due to rifampicin combination [25,26,30,31]. In a prospective report of MRSA native valve endocarditis, rifampicin-vancomycin therapy resulted in non-significantly prolonged bacteraemia as compared to vancomycin only [25]. A retrospective study investigated the benefit of adjunctive rifampicin therapy in native valve endocarditis with a high MRSA prevalence (76%) and showed prolonged bacteraemia and a significant negative prognostic impact for patients receiving rifampicin therapy [26]. Delay in effective antibiotic therapy may result in worse outcome in SAB [35]. MRSA has been associated with delayed effective antibiotic therapy and worse prognosis as compared to MSSA [2]. The present study included only MSSA cases and 99% of patients received effective intravenous antibiotic therapy from the first day of the blood culture collection. Invasive and bacteraemic infections due to MRSA are rare in Finland with MRSA prevalence remaining near 3% [36]. Vancomycin has been shown to increase the tendency for bacteraemia persistence and recurrence as compared to antistaphylococcal penicillin cloxacillin [37]. Vancomycin was provided to only 2% of our patients. Thus, the impact of rifampicin therapy on outcome could be evaluated without disturbance from delayed empirical antibiotic therapy. However, our study gives no answer on the value of rifampicin combination therapy in MRSA bacteraemia.

No data is available on the optimal timing of adjunctive rifampicin therapy initiation alongside standard antibiotic treatment. In MRSA bacteraemia, early rifampicin treatment was associated with emergence of resistance in 37–56% of cases when rifampicin was started during the bacteraemic phase [26,30,31]. In one study, each case of rifampicin resistance was due to therapy started during the bacteraemic phase whereas no resistance developed when rifampicin was initiated subsequent to bacteraemia clearance [26]. One report compared MRSA and hVISA bacteraemic patients who were receiving rifampicin-vancomycin therapy and demonstrated prolonged bacteraemia and higher rifampicin-resistance development for hVISA cases. Due to hVISA, the vancomycin serum concentration was below the required hVISA MIC and the authors interpreted this as rifampicin monotherapy resulting in the development of rifampicin resistance [30]. Hence, the development of rifampicin resistance in MRSA bacteraemia might be related to relative weak anti-staphylococcal effect or problems in tissue penetration of vancomycin or to longer persistence of bacteraemia during vancomycin therapy rather than due to methicillin-resistance of the staphylococcal strain [37,38]. Follow-up blood cultures were not routinely taken in our patient cohort and the present study does not give an answer on the question of possibility of rifampicin resistant development in MSSA bacteraemia.

In the present study, the median time from blood culture to clinical awareness of *S*. *aureus* being the causative pathogen was 3 days in average. In practice, rifampicin treatment was commenced earliest at this time point i.e. 3 days subsequent to effective empirical antimicrobial therapy, mostly a beta-lactam. Our results clearly point to a positive prognostic impact when rifampicin treatment is started during the first week of positive blood culture. Altogether the present study included 26 patients with a deep infection focus in which rifampicin was started later than 7 days past positive blood culture and continued for longer than 14 days. Among these patients rifampicin treatment showed no signs of improved prognosis. However, these results have to be interpreted with great caution due to the small patient number (n = 26) and the retrospective nature of this study. Once started, rifampicin therapy is usually continued for several weeks [38]. The patient group who received rifampicin therapy shorter that 14 days was also too small (n = 50) for rigorous and detailed analysis. Thus, no firm conclusions and no recommendations concerning a lack of prognostic influence of short rifampicin therapy or late started rifampicin therapy (≥14 days) can be made. Future randomized controlled trials with higher patient numbers are needed both to confirm our findings and to give an answer to whether later onset or shorter rifampicin treatment will have any prognostic impact.

The present study did not investigate the impact of different rifampicin dosages or the impact of administration routes on prognosis. Rifampicin was administered 450 mg once daily for patients under 50 kg and 600 mg once daily for patients over 50 kg in weight. This dosage, however, is far lower than that used in some studies e.g. 450 mg every 12 hours [11] or 20mg/kg given in divided doses twice a day without exceeding daily doses of 1800 mg [14]. The potential positive prognostic impact associated with adjunctive rifampicin therapy for at least 14 days observed in the present study might have been stronger with higher rifampicin doses.

The present study excluded patients with a fatal outcome within 3 days in order to allow for death before positive blood culture results and the possibility to receive rifampicin therapy. It could be argued that patients who died early were more ill and thus more likely to be treated without rifampicin. However, as a subanalysis the Cox regression was performed by excluding patients with a fatal outcome within 21 days and the results were very similar to the main Cox regression analysis with early onset of rifampicin therapy for at least 14 days associating to positive prognosis.

Also other attempts were made to control reasons for the difference in outcome between the groups. Most of the factors with prognostic impact in this study have been identified earlier e.g. age [3,39,40], McCabe′s classification [3], endocarditis [3,39] and IDS consultation [3,6]. Patients treated without rifampicin therapy were less often males and had more often healthcare-associated SAB. The present study can not provide any clear explanation for this gender distribution. Healthcare-associated SAB has been associated to poorer SAB outcome [39], however, when controlling for all of these factors, early onset rifampicin therapy for at least 14 days still remained a favourable prognostic factor (OR 0.33, p<0.01). Case fatality in our material was only 18% at 90 days follow-up which is at the lower end compared to many other SAB studies [3,40,41,42]. The low mortality has most probably decreased the power to detect a positive prognostic effect of rifampicin.

SAB patients with alcoholism and acute or chronic liver diseases were excluded from our main patient cohort. The risk for liver failure as a complication for rifampicin therapy is strongly accentuated in patients with alcoholism or liver diseases and these conditions are viewed as contraindications for rifampicin [43]. Alcoholism and liver diseases unavoidably creates a statistical bias as patients with these conditions are very unlikely to receive rifampicin therapy.

In conclusion, despite the retrospective nature of the present study the results indicated that in MSSA bacteraemia patients with a deep infection focus may gain from adjunctive rifampicin therapy of at least 14 days of length when rifampicin is initiated within 7 days of positive blood culture. Recommendations on rifampicin combination with other antimicrobial regimens in MRSA bacteraemia cannot be made based on the results of the present study and prospective randomized studies on the effect of rifampicin combination in MSSA and MRSA bacteraemia are needed.
